# Mirizzi Syndrome and Its Surgical Interventions: A Case Report

**DOI:** 10.7759/cureus.66680

**Published:** 2024-08-12

**Authors:** Saili Kelshikar, Virendra Athavale, Rushabh A Parekh

**Affiliations:** 1 General Surgery, Dr. D. Y. Patil Medical College, Hospital and Research Centre, Dr. D. Y. Patil Vidyapeeth, Pune (Deemed to be University), Pune, IND

**Keywords:** biliary-enteric anastomosis, cholecystobiliary fistula, subtotal cholecystectomy, obstructive jaundice, choledocholithiasis, cholelithiasis, mirizzi syndrome

## Abstract

A rare side effect of cholelithiasis, called Mirizzi syndrome (MS), arises when gallstones that are impacted in the Hartmann’s pouch or the cystic duct extrinsically compress the common bile duct. This condition is typically managed with a cholecystectomy. In this case report, different surgical approaches are described according to each type of Mirizzi. We report a 62-year-old female who presented with abdominal pain. She underwent endoscopic retrograde cholangiopancreaticography (ERCP) and was diagnosed with MS. We performed a subtotal cholecystectomy with a choledochoduodenostomy.

## Introduction

An uncommon side effect of cholelithiasis is Mirizzi syndrome (MS). Lodged gallstones in the Hartmann's pouch or the cystic duct can lead to this condition [1]. These stones constrict the common bile duct (CBD) externally, which may cause symptoms of obstructive jaundice [2-4]. In 1905, it was noted by Kehr, a German physician, and later, Pablo Mirizzi, an Argentinian surgeon, called it síndrome del conducto hepático [5].

The presentation of MS can be vague, which makes it difficult to diagnose [3,4]. It appears to have a female preponderance [1,6]. MS is rare, but in underdeveloped countries, the documented prevalence is as high as 5.7% [7].

The first-line radiological investigation of choice is an ultrasound; however, magnetic resonance cholangiopancreatography (MRCP) can be very useful for diagnosis. Early intervention is necessary to avoid complications. The treatment is a total or subtotal cholecystectomy [3,6]. Laparoscopy is beneficial, but the conversion rate to open surgery is high, which can lead to complications [2].

## Case presentation

A 62-year-old female came to the Outpatient Department with chief complaints of right upper abdominal pain for 10 days. The pain was moderate and radiated to the back. It was aggravated by the intake of food and relieved by taking painkillers. She also complained of jaundice for 10 days. It was not associated with nausea, vomiting, fever, bowel abnormalities, itching, or loss of appetite. Examination revealed icterus and a soft abdomen with tenderness over the right hypochondrium.

**Figure 1:**
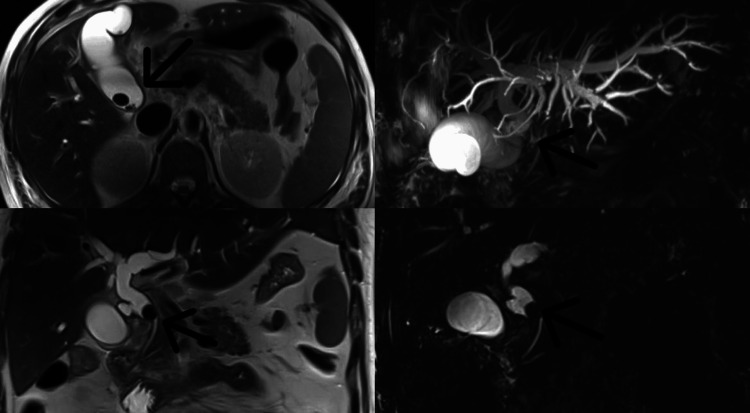
MRCP films Arrows pointing show multiple well-defined hypointense filling defects in the common bile duct (CBD) and the common hepatic duct (CHD). There is dilatation of the proximal hepatic biliary radicals, right and left hepatic ducts, the CHD, and the CBD. A calculus is noted in the neck of the gall bladder, causing compression of the CBD, suggestive of Mirizzi syndrome. MRCP: Magnetic resonance cholangiopancreaticography

MRCP (Figure 1) showed multiple well-defined hypointense filling defects in the CBD and the common hepatic duct (CHD), the largest measuring 14 mm. There was dilatation of the proximal hepatic biliary radicals, right and left hepatic ducts, the CHD (12 mm), and the CBD (10 mm). The gall bladder was partially distended, with an edematous, inflamed wall of 10 mm thickness and mild fat stranding in the gall bladder fossa, which was suggestive of cholecystitis. A calculus, approximately 15 mm in size, was noted in the neck of the gall bladder, causing compression of the CBD, suggestive of MS. There was evidence of continuity seen in the neck of the gall bladder and CBD region, suggesting fistula formation.

**Figure 2:**
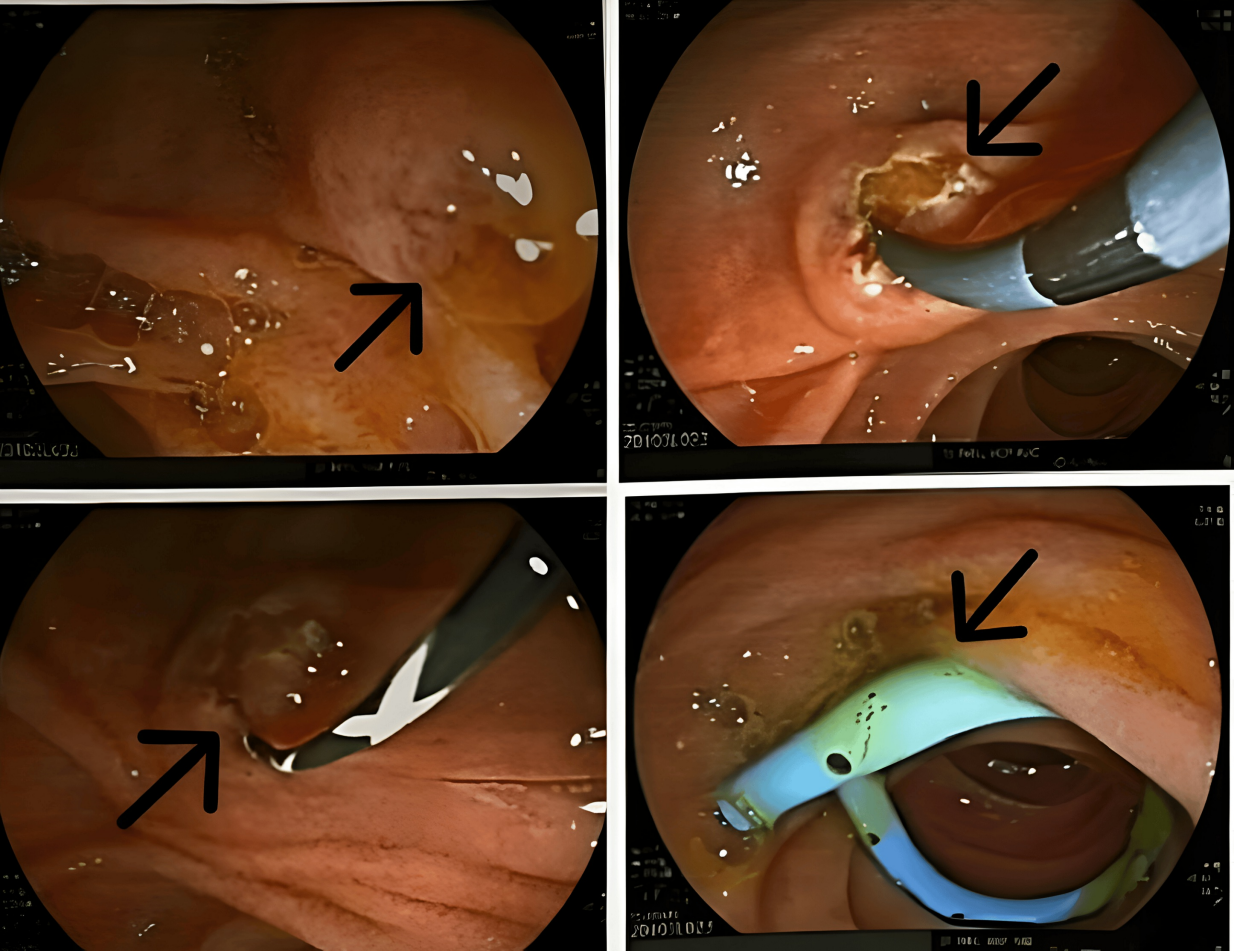
ERCP findings Arrows show a dilated common bile duct and point at the stent placed in the common bile duct. ERCP: Endoscopic retrograde cholangiopancreaticography

The patient was planned for endoscopic retrograde cholangiopancreatography (ERCP) (Figure 2) to confirm the diagnosis and to relieve biliary obstruction. It was suggestive of multiple filling defects in the biliary tree, the greatest of which was near the cystic duct's insertion, as well as a dilated CBD (16 mm). A biliary sphincterotomy was done, and a plastic stent was placed in the CBD, but complete CBD clearance could not be achieved.

**Figure 3:**
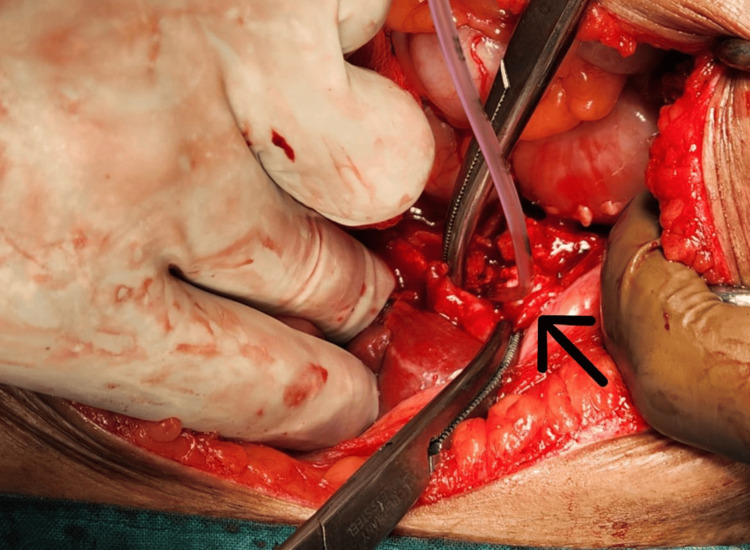
Intraoperative photo The arrow shows cannulation of the common bile duct is done from inside through a fundus first approach.

**Figure 4:**
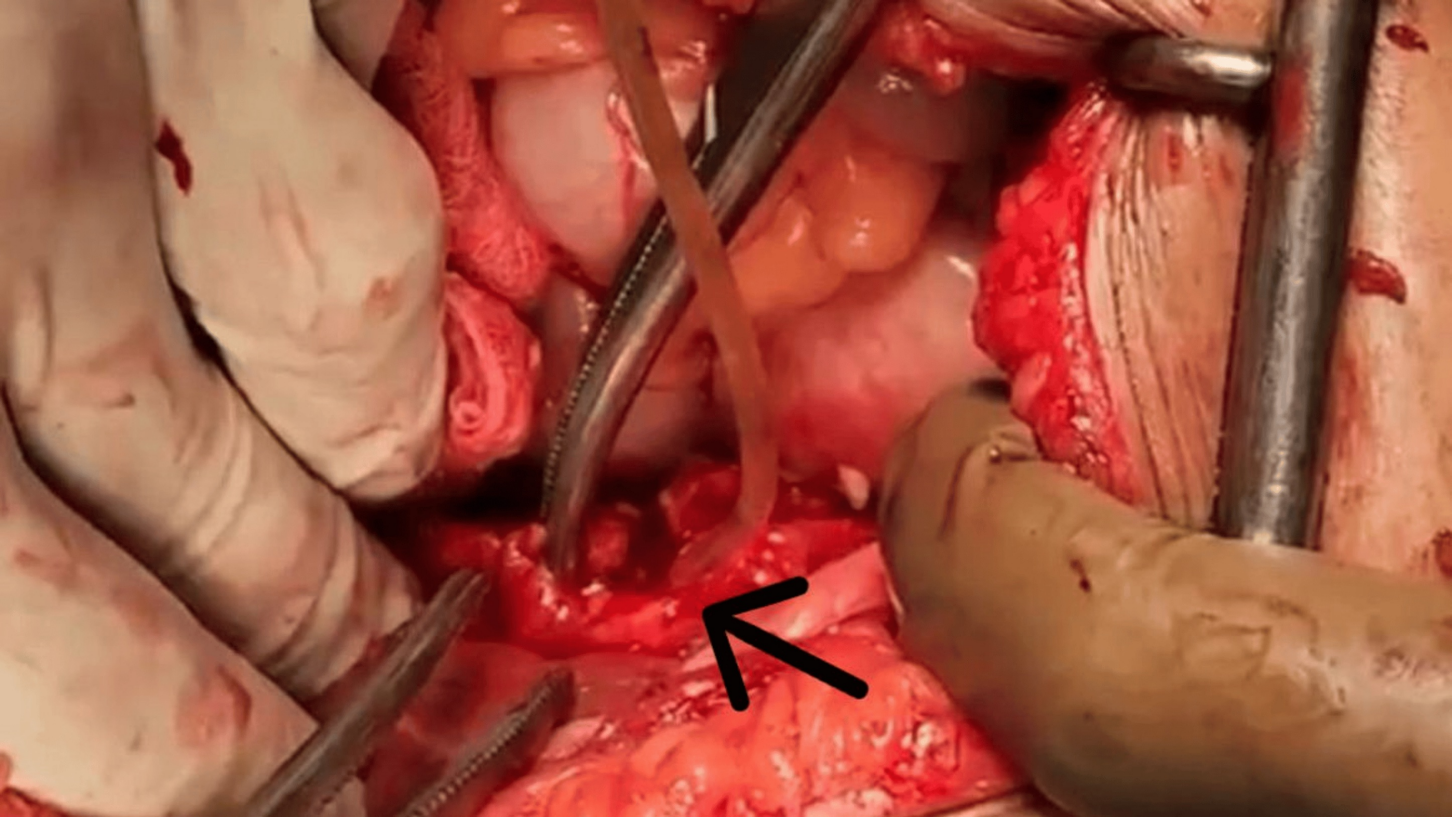
Intraoperative photo The arrow shows confirmation of a cholecysto-biliary fistula due to the bile reflux post-cannulation.

**Figure 5:**
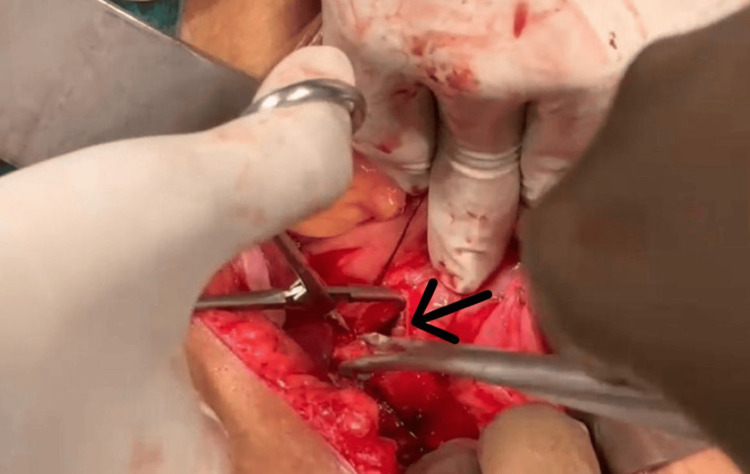
Intraoperative photo The arrow shows gallstones being retrieved from the common bile duct.

**Figure 6:**
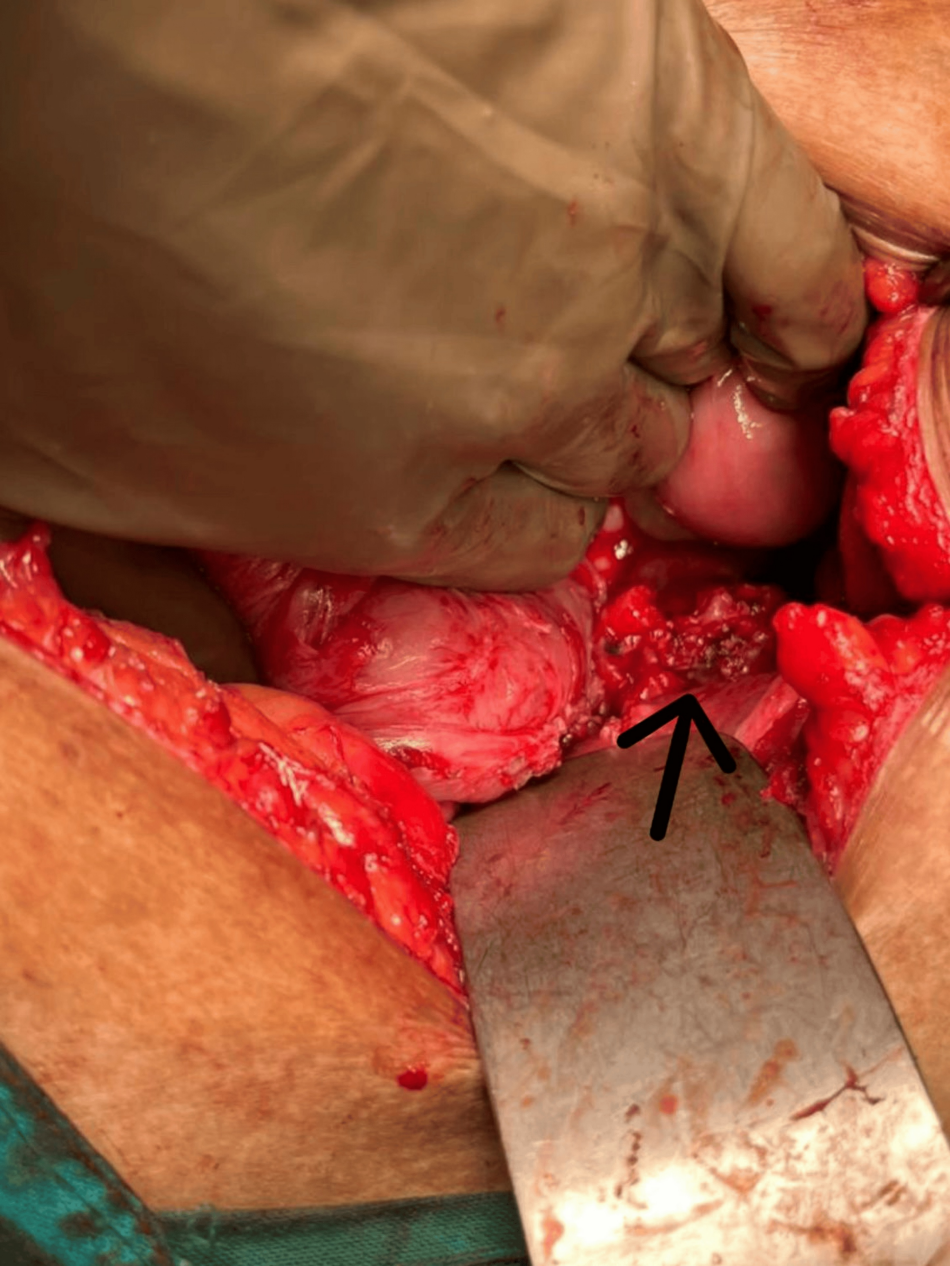
Intraoperative photo The arrow shows a stump of the gall bladder after a subtotal cholecystectomy.

The patient was scheduled for a cholecystectomy along with CBD exploration. We decided to proceed with a subtotal cholecystectomy through a fundus-first approach because there were signs of cholecystitis. The cystic duct was explored from the inside. A fistula was evident, which, when cannulated (Figure 3), showed bile reflux (Figure 4), confirming a cholecysto-biliary fistula. Gallstones were retrieved (Figure 5). The gall bladder stump was closed (Figure 6).

**Figure 7:**
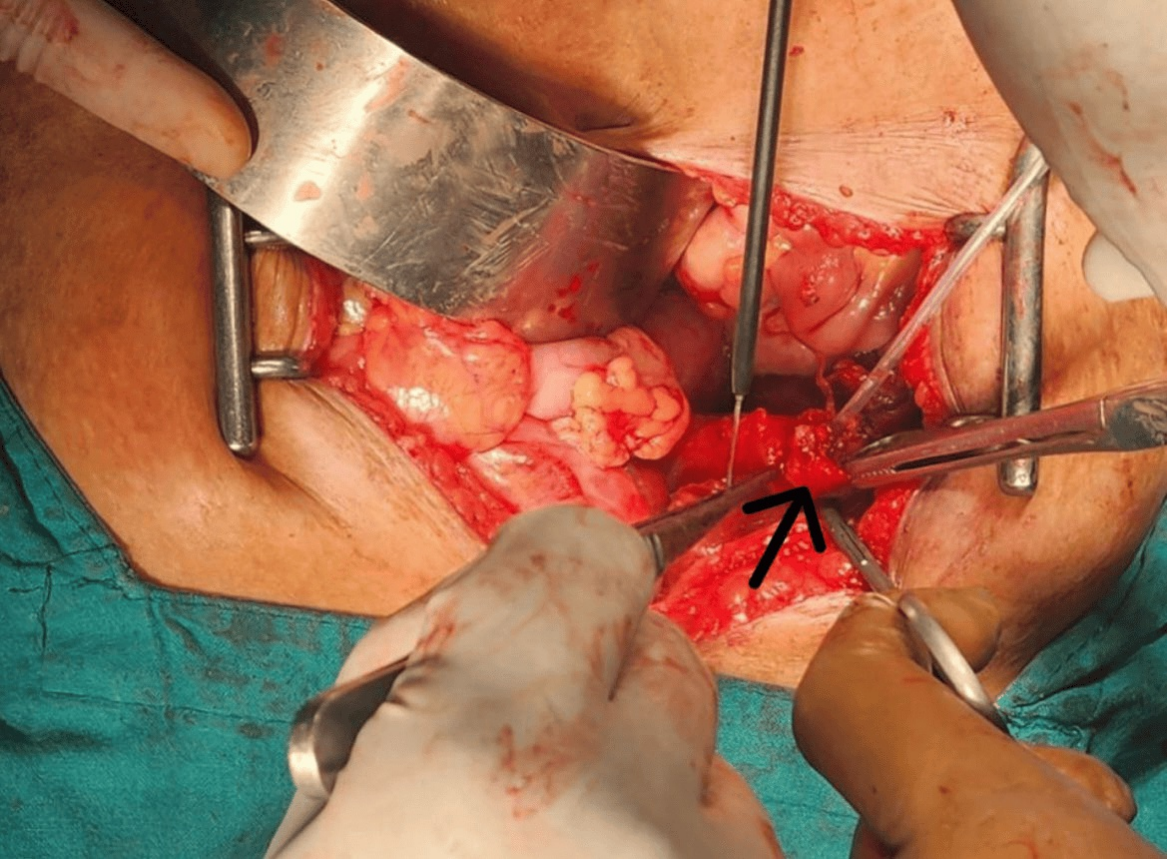
Intraoperative photo The arrow shows cannulation of the common bile duct done through a separate incision.

**Figure 8:**
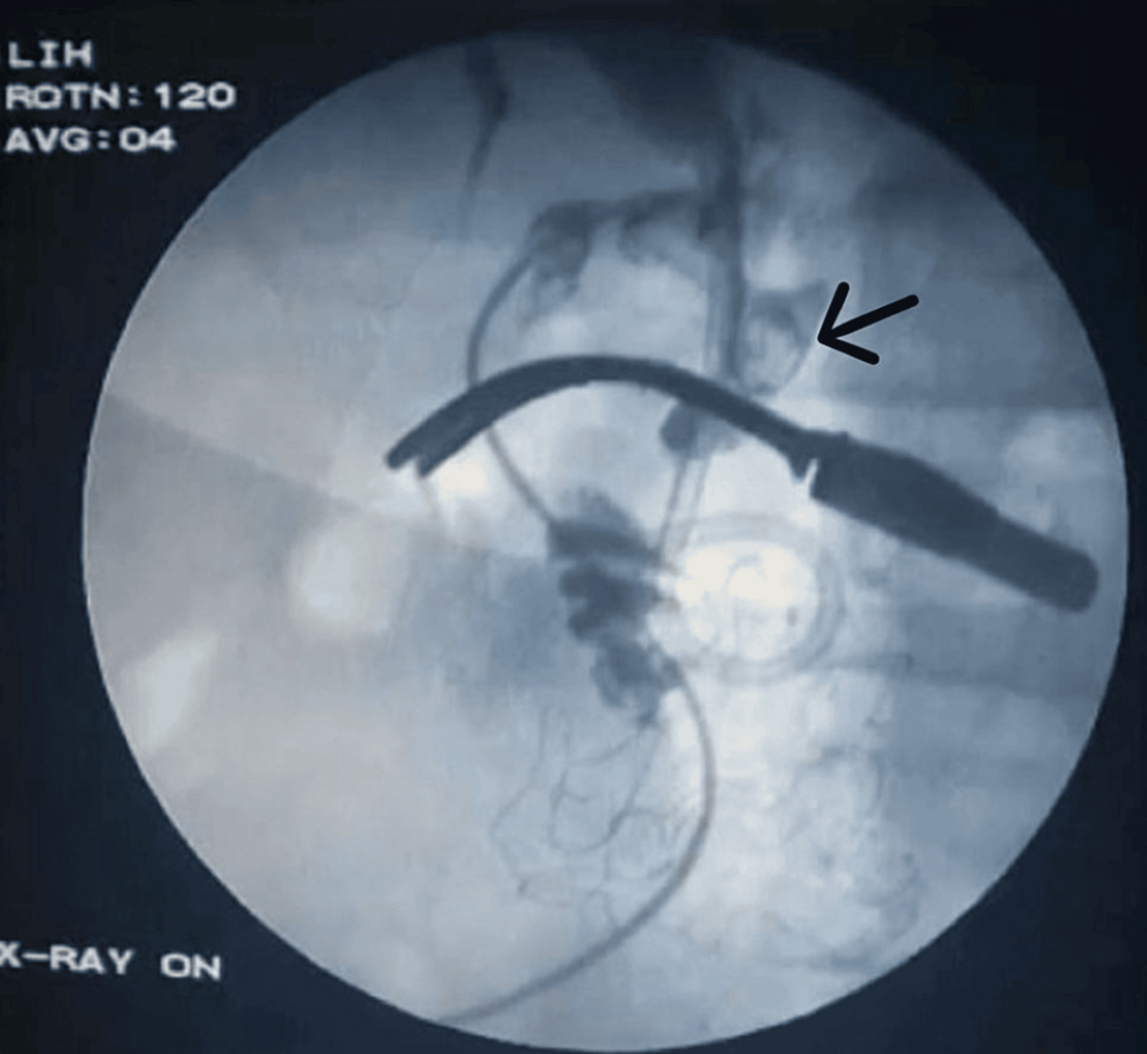
Intraoperative cholangiogram The arrow points at the common bile duct with multiple defects.

The CBD was explored through a separate incision and cannulated (Figure 7). There was evidence of inflammation. The CBD showed multiple filling defects on an intraoperative cholangiogram (Figure 8). The CBD was flushed, residual stones were retrieved, and complete ductal clearance was achieved.

**Figure 9:**
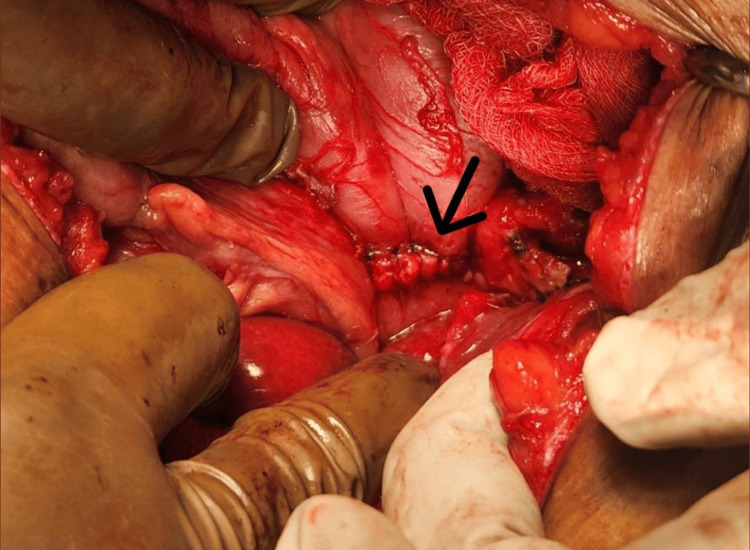
Intraoperative photo The arrow shows biliary enteric anastomosis between the duodenum and the common hepatic duct.

**Figure 10:**
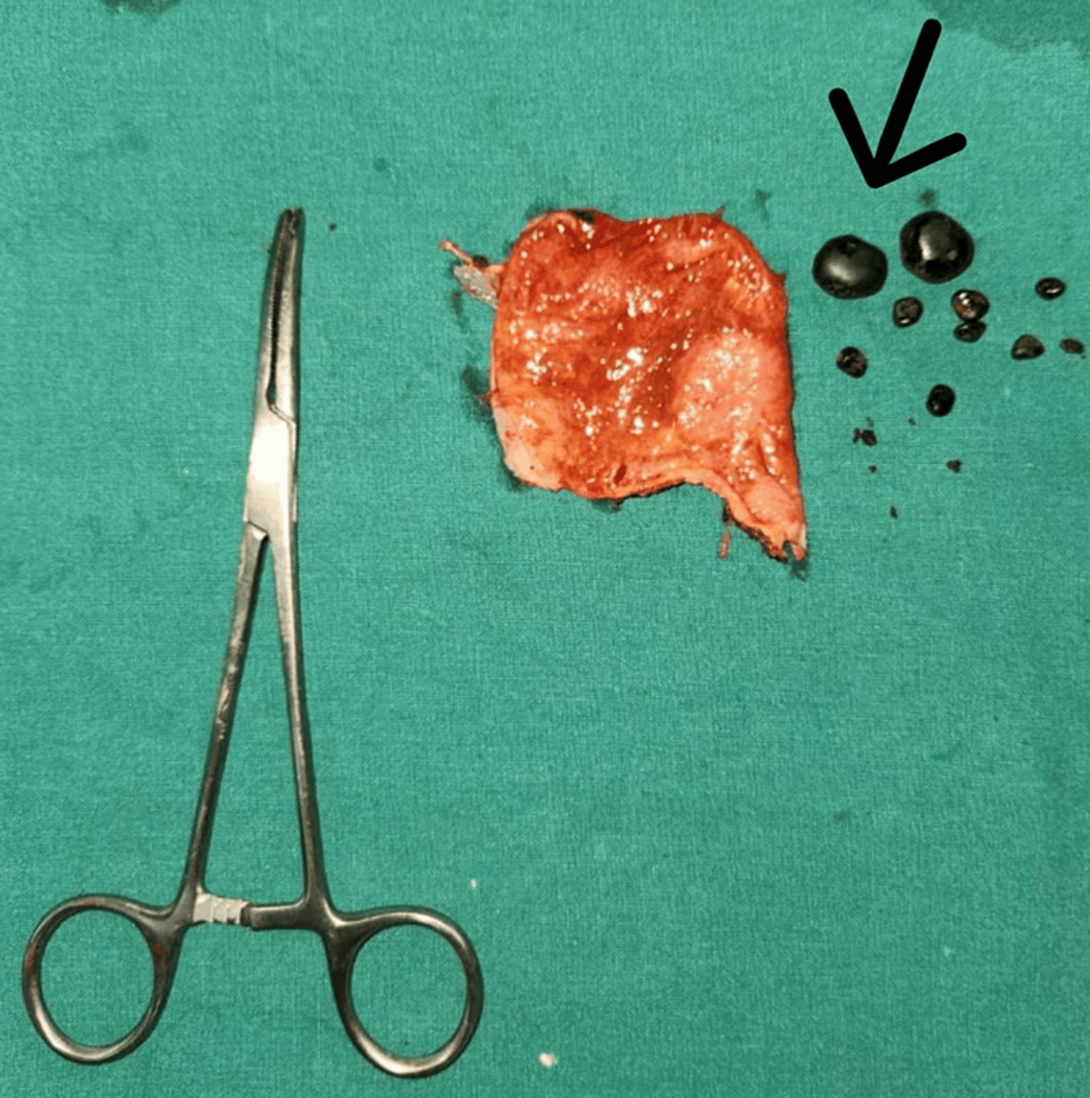
Specimen photo The arrow shows an excised portion of the gallbladder with retrieved gallstones after a subtotal cholecystectomy.

Since there was a presence of a cholecysto-biliary fistula, and the gall bladder was inflamed, we decided to go for a bilio-enteric anastomosis. The duodenum was mobilized to the CHD. A choledocho-duodenostomy was performed (Figures 9-10).

## Discussion

The presentation of MS varies clinically, ranging from asymptomatic to non-specific. The most typical presentation is obstructive jaundice, which is followed by high liver enzymes, pain in the right upper abdomen, and other symptoms like nausea, vomiting, fever, diarrhea, and constipation. MS may rarely present as gallstone ileus [5,6]. 

McSherry classified MS on the basis of ERCP findings of fistula formation. In this classification, Type 1 shows impacted gallstones pressing upon the cystic duct or the Hartmann’s pouch, with external compression on the CBD. Type 2 entails the formation of a cholecysto-biliary fistula as a result of stone erosion into the CBD [3].

Csendes further divided the cholecysto-biliary fistulas into three categories, depending on the degree of the CBD circumferential erosion. After revision, Type 2 had erosions less than one-third of the CBD’s circumference. Up to two-thirds of the CBD circumference was eroded in Type 3. Type 4 had the wall of the CBD completely destroyed [2].

In addition to these classifications, Beltran described a Type 5, which is categorized into Type 5A, any combination of the first four types with the development of a cholecysto-enteric fistula but not with gallstone ileus, and Type 5B, with gallstone ileus [4].

The management of the majority of cases of MS is surgical, although this is difficult because preoperative diagnosis is frequently overlooked. The anatomy is distorted as a result of severe inflammation, with dense adhesions and associated edematous tissues. Depending on the severity of the lesion, operative mortality and postoperative morbidity increase [2,5,8]. When dissecting Calot's triangle, the possibility of bile duct damage or severe bleeding is increased if a fistula is present. In addition, infections, cutaneous fistulas, delayed biliary strictures, secondary biliary cirrhosis, and even mortality can result from inflammation [3,5,7,8].

MS Type 1 can often be managed with a classic cholecystectomy. However, in cases with severe inflammation of Calot’s triangle, a subtotal cholecystectomy might be the best option [5,8,9]. Partial resection of the gallbladder is recommended to aid in the removal of gallstones and to visualize the cholecysto-biliary fistula [2,6]. The gallbladder is accessed through the fundus, and gallstones that are loosely attached are easily removed, while those adhered to the wall are extracted with the mucosa or the entire wall. The cystic duct is examined from within the opened gallbladder [9].

It is vital to investigate the CBD because choledocholithiasis is frequently associated with MS. To lower the risk of bile duct stricture and bile leak, a secondary incision is made, and a Kehr tube is introduced distal to the fistula. Using the tube, an intraoperative cholangiography should be carried out. In the event that no fistula is seen, the gallbladder stump is closed over the bile duct, and a partial cholecystectomy is carried out [5,10].

Dissection is done for MS Type 2, starting at the gallbladder fundus and moving toward Hartmann's pouch. Bile reflux confirms the existence of a cholecysto-biliary fistula since the cystic duct is typically obstructed. A subtotal cholecystectomy is performed, leaving part of the gallbladder wall around the fistula to repair the bile duct [8].

A subtotal cholecystectomy is also carried out in cases of MS Type 3, leaving part of the gallbladder wall in place to seal the bile duct. Nonetheless, a bilio-enteric anastomosis to the duodenum or jejunum is necessary if the gallbladder wall is inflamed [5,8,10].

The preferred method of managing MS Type 4 with persistent gallbladder inflammation is a Roux-en-Y hepaticojejunostomy, as it can lead to strictures and larger fistulas [9].

Dividing and closing the bilioenteric fistula across the duodenum, stomach, colon, or small bowel is the treatment for MS Type 5A. A partial or total cholecystectomy may be required, depending on whether there is bile duct compression or a cholecysto-biliary fistula present. It is recommended that, in MS Type 5B, the acute condition of the accompanying gallstone ileus be treated first, and then the gallbladder after three months [4,5].

Although the minimally invasive laparoscopic approach has benefits, conversion rates to open surgery are disappointingly high. It is recommended to limit the laparoscopic approach to managing Type I only, due to the increased risk of bile duct injury because of severe inflammation [6].

## Conclusions

MS should be considered as a differential diagnosis in patients who present with acute biliary colic and obstructive jaundice, due to the challenge it poses, which complicates a surgery that is assumed to be straightforward. After establishing the correct diagnosis, surgery should be performed to identify and manage the type of MS adequately. This case report helps review the different surgical approaches according to each type of Mirizzi.
